# A study on the risk prediction model for venous thromboembolism in orthopedic inpatients based on machine learning

**DOI:** 10.3389/fmed.2025.1574546

**Published:** 2025-06-26

**Authors:** Bo Zhang, Yumei Qin, Liandi Jiu, Chunming Qin, Jiangbo Wang, Haiqing Zhao

**Affiliations:** ^1^Digital Health China Technologies Co., Ltd., Beijing, China; ^2^Nanxishan Hospital of Guangxi Zhuang Autonomous Region, The Second People’s Hospital of Guangxi Zhuang Autonomous Region, Guilin, China

**Keywords:** venous thromboembolism, machine learning, risk assessment, orthopedic inpatients, clinical decision support

## Abstract

**Objective:**

To construct a venous thromboembolism (VTE) risk prediction model for orthopedic inpatients using machine learning modeling techniques, identify high-risk patients, and optimize clinical interventions.

**Methods:**

This study involved a retrospective analysis of 286 orthopedic inpatients from Nanxishan Hospital of Guangxi Zhuang Autonomous Region (The Second People’s Hospital of Guangxi Zhuang Autonomous Region) from January 1, 2022 to December 31, 2022. To ensure patient information security, all data were fully anonymized before access. The collected data included basic information such as gender, age, ethnicity, and body mass index (BMI), lifestyle factors and medical history (including smoking, alcohol use, diabetes, hypertension, and personal and family history of VTE), clinical test results (such as thrombin time, plasma D-dimer, total bilirubin, and urinary protein via dry chemistry), as well as genetic test results related to VTE risk. Feature analysis and data mining were conducted, and eight different machine learning algorithms were used to build the prediction model. The SHapley Additive exPlanation (SHAP) method was used to rank the feature importance and explain the final model.

**Results:**

Through a comprehensive evaluation and comparison of eight different machine learning models, the results clearly indicate that the XGBoost model outperforms the others across all performance metrics, achieving the highest accuracy of 0.828 and AUROC of 0.931, significantly surpassing the other models, particularly in prediction accuracy and discriminative ability. Compared to the traditional Caprini scoring model, XGBoost not only shows improvements in accuracy and specificity but also demonstrates a significant increase in Area Under the Curve (AUC), further validating its superior performance in VTE risk prediction.

**Conclusion:**

This model can be effectively used for early risk prediction of VTE, helping to reduce the incidence of venous thromboembolism in orthopedic patients. Given its promising results, further validation and wider application of the model in clinical settings are warranted to enhance patient outcomes and improve preventive strategies.

## Introduction

Venous thromboembolism (VTE), which includes deep vein thrombosis and pulmonary embolism, is the third most common cardiovascular disease worldwide, following myocardial infarction and stroke (1, 2). VTE is especially prevalent among hospitalized patients. In China, studies show that as many as 45.2% of hospitalized patients are at high risk for VTE, with 53.4% of surgical patients facing elevated risk (3). Orthopedic patients, in particular, are at significantly higher risk due to factors such as surgery, prolonged immobility, and common comorbidities (4–6). This puts them at a higher incidence and mortality rate for VTE, placing a considerable physical and economic burden on both patients and their families. Despite the availability of effective preventive measures, including anticoagulation therapy and mechanical prevention, the incidence of VTE remains high (7, 8). Given its high mortality rate and severe complications, early identification and accurate assessment of VTE risk, followed by personalized prevention strategies, is a critical clinical challenge. Therefore, research on VTE risk prediction for orthopedic inpatients is of urgent importance.

Currently, VTE risk assessment primarily relies on clinical experience and standardized scoring tools such as the Caprini, Padua, and Khorana scores (9–11). These scales evaluate risk based on a range of known factors, including age, gender, body mass index (BMI), and comorbidities. The Caprini score, in particular, is widely used for assessing VTE risk in surgical patients, especially in orthopedic inpatients (12–14). However, despite their widespread use, these scoring systems have limited value in guiding specific preventive measures and often fail to fully account for individual patient differences and the complexity of clinical situations.

In recent years, with the rapid development of artificial intelligence, machine learning has made significant progress in various fields (15) such as disease risk prediction (16), drug dosage individualization (17), and treatment outcome evaluation (18). Machine learning can handle complex nonlinear relationships and extract potential key factors from vast amounts of data, thereby improving prediction accuracy. At the same time, the widespread use of Electronic Medical Record (EMR) systems in hospitals has made the collection of clinical data more precise and convenient, providing reliable data support for machine learning modeling. As a result, machine learning methods based on EMRs have gradually gained the attention and recognition of clinicians (19, 20).

In this study, we aimed to develop and validate explainable machine learning models for early and accurate prediction of VTE in orthopedic inpatients by analyzing their clinical characteristics, medical history, laboratory results, and genetic testing data. Based on this risk assessment, appropriate interventions will be implemented according to different risk stratifications to reduce the incidence of VTE-related complications. Through predictive analysis using machine learning, we aim to improve clinical outcomes, optimize healthcare resource utilization, enhance patient safety, and improve the quality of care.

In conclusion, personalized VTE risk assessment tools represent a significant advancement in the management of surgical patients. By integrating modern machine learning technologies, we aim to bridge the gap between traditional risk assessment methods and the needs of high-risk patients, supporting precision medicine and individualized care. The findings of this study have the potential to transform current VTE management practices, making them more aligned with patients’ specific needs, and driving the medical field toward a more precise and efficient future.

## Methods

### Study population

This is a single-center retrospective cohort study, with subjects consisting of 286 orthopedic inpatients at Nanxishan Hospital of Guangxi Zhuang Autonomous Region (The Second People’s Hospital of Guangxi Zhuang Autonomous Region) from January 1, 2022 to December 31, 2022. The inclusion criteria included the following: (1) aged 18 years or older; (2) orthopedic inpatients with a hospital stay > 3 days; (3) completed VTE risk gene polymorphism assessment; (4) no contraindications to anticoagulation; (5) voluntarily agreed to participate in the study and signed an informed consent form. The exclusion criteria were as following: (1) patients who were bedridden or had restricted mobility (e.g., hemiplegia) prior to admission; (2) patients with renal or hepatic dysfunction; (3) patients with hematologic disorders or coagulation dysfunction; (4) pregnant or breastfeeding women; (5) patients with severely missing gene or clinical phenotype data. After applying these criteria, the final study cohort was selected, ensuring the representativeness and scientific rigor of the research findings.

### Data collection and processing

We collected demographic information (such as gender, age, race, BMI), lifestyle factors and medical history (including smoking, alcohol consumption, diabetes, hypertension, VTE history, family history of VTE), laboratory test results (such as thrombin time, plasma D-dimer, total bilirubin, urinary protein by dry chemistry, etc.), as well as genetic polymorphism data to identify and select features associated with VTE and construct a risk prediction model. All data were obtained from the EMR system.

Firstly, features with more than 40% missing values were excluded from subsequent analyses to minimize potential bias. A total of 34 features, including age, body mass index (BMI), sex, and others, were ultimately retained and missing data were addressed using median imputation. Outliers for each feature were identified using the IQR (Interquartile Range) method and replaced with the corresponding feature’s median value. Finally, Min-Max scaling was applied to normalize the data, rescaling it to a range of 0 to 1.

### Feature selection

Selecting the most relevant and impactful features from the original dataset not only improves model performance and interpretability but also reduces storage and computational resource requirements.

Firstly, this study identified the optimal feature subset using Recursive Feature Elimination (RFE), based on Random Forest model and XGBoost model, to reduce dimensionality and improve model performance. Subsequently, by comparing the differences in each feature between the VTE group and the non-VTE group, features without significant differences (*p* < 0.1) were excluded.

**Figure 1:**
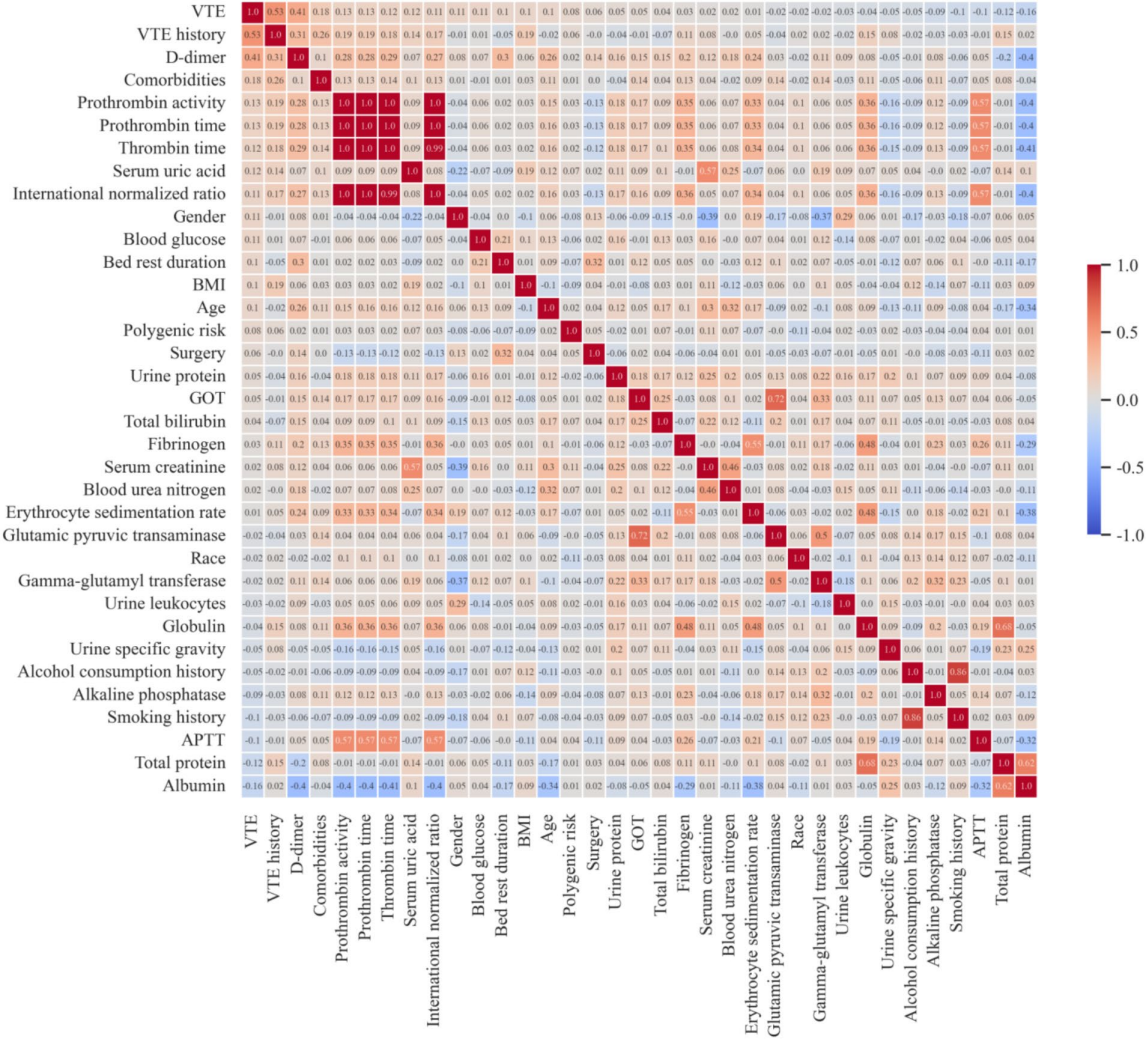
Feature correlation heatmap. VTE, venous thromboembolism; BMI, body mass index; GOT, glutamic-oxaloacetic transaminase; APTT, activated partial thromboplastin time.

Additionally, due to the potential impact of multicollinearity among features on prediction accuracy, when two features were highly correlated (correlation coefficient > 0.9) in Spearman’s correlation analysis, the feature less correlated with the outcome was eliminated from the subset, as shown in Figure 1. Finally, combining insights from VTE-related literature, the final features used to construct the model were determined.

### Model construction and selection

To develop a VTE risk prediction model, this study randomly selected 80% of the dataset as the training set for model training, while the remaining 20% was used as the test set for model performance evaluation (internal validation). To ensure class balance between positive and negative samples in the training set, Synthetic Minority Oversampling Technique (SMOTE) was applied to the training data.

A total of eight binary classification machine learning models were constructed to predict the VTE risk in orthopedic inpatients, including Naive Bayes (NB), K-Nearest Neighbors (KNN), Support Vector Machines (SVM), Logistic Regression (LR), Decision Tree (DT), Adaptive Boosting (AdaBoost), eXtreme Gradient Boosting (XGBoost), and Random Forest (RF).

To enhance model robustness and reduce the risk of overfitting, five-fold cross-validation was employed for training. Model performance was comprehensively evaluated using various metrics, including Accuracy, Sensitivity, Specificity, Precision, F1 Score, and AUC. The XGBoost model and Random Forest model, recognized as the two best-performing predictive model, is utilized for further model optimization.

### Hyperparameter tuning and internal validation

Hyperparameter tuning can optimize the performance and generalization ability of the model. A combination of random search and manual fine-tuning was employed to optimize hyperparameters, ensuring the model achieved its best performance.

An internal data set consisting of 58 samples, including 41 negative cases and 17 positive cases was employed for the internal validation.

### Model explanation

To enhance the transparency and reliability of the risk assessment model, this study uses SHAP (SHapley Additive exPlanations) to interpret the model’s predictions. SHAP is effective because it provides both a broad overview of feature importance and a detailed explanation of individual predictions. At the global level, SHAP evaluates the contribution of each feature across all samples, highlighting the most influential factors driving the model’s decisions. This insight aids in model optimization and identifying key features. At the local level, SHAP shows how each feature influences a specific prediction. This helps clarify how particular factors impact an individual patient’s risk score, providing valuable guidance for targeted interventions.

### Statistical analysis

Continuous variables are presented as mean (standard deviation), while categorical variables are shown as counts and percentages. For the comparative analysis between negative and positive subgroups, continuous variables were assessed using the Mann–Whitney U test or T-test, and categorical variables were analyzed using the Chi-square test. Spearman correlation analysis was used to evaluate the relationships between continuous variables. The predictive power was assessed using the Area Under the Curve (AUC). All data analyses were conducted using Python 3.8.3 and the scikit-learn library (version 1.3.2). The corresponding source code is publicly available on GitHub at https://github.com/LDjiu/VTE_predict.

## Results

### Patient characteristics

**Figure 2:**
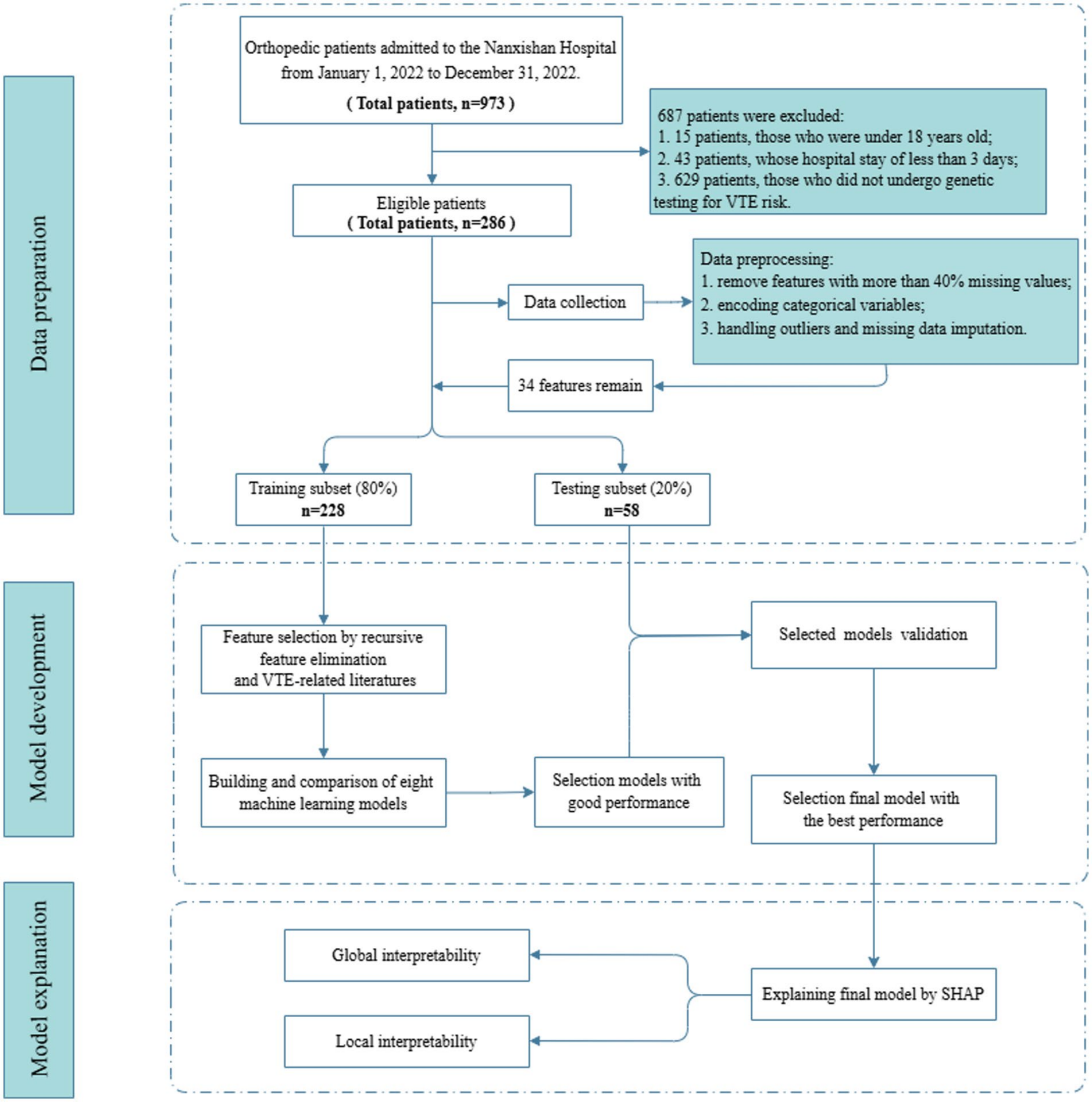
Flow diagram for patients screening, data processing, model development and model explanation.

This retrospective study included 286 patients, comprising 86 patients who developed VTE during hospitalization and 200 patients who did not. Among 973 patients admitted to the orthopedic department of Nanxishan Hospital from January 1, 2022 to December 31, 2022, 687 patients were excluded, including 15 patients who were under 18 years old, 43 patients whose hospital stay of less than 3 days, and 629 patients who did not undergo genetic testing for VTE risk. The 286 patients were divided into independent training and testing sets. The detailed design of the study was shown in Figure 2.

Demographic characteristics, lifestyle habits, medical history, and laboratory test results were collected. Features with more than 40% missing values were removed, leaving 34 features. A comparison of these 34 features between VTE and non-VTE patients is detailed in Table 1. Continuous variables were described using mean (standard deviation), and categorical variables were described using frequencies. Significant differences (*p* < 0.05) were observed between VTE and non-VTE patients in terms of VTE history, comorbidities, total protein, albumin, D-dimer, and erythrocyte sedimentation rate.

**Table 1:** Comparison of demographic and clinical characteristics and outcomes between non-VTE and VTE patients.

Variables	Non-VTE (*n* = 200)	VTE (*n* = 86)	*P*-value
Polygenic risk^*^, *n* (%)			0.326
Low risk	25 (12.5)	6 (7.0)	
Median risk	118 (59.0)	51 (59.3)	
High risk	57 (28.5)	29 (33.7)	
Gender, Male, *n* (%)	105 (52.5)	35 (40.7)	0.089
Race, Han Chinese, *n* (%)	181 (90.5)	79 (91.9)	0.887
Age, year, mean (SD)	63.9 (14.0)	67.1 (12.9)	0.066
BMI, kg/m^2^, mean (SD)	23.1 (3.2)	23.7 (3.0)	0.138
Smoking history, *n* (%)	11 (5.5)	1 (1.2)	0.116
Alcohol consumption history, *n* (%)	9 (4.5)	2 (2.3)	0.514
VTE history, *n* (%)	3 (1.5)	35 (40.7)	<0.001
Comorbidities, *n* (%)	12 (6.0)	15 (17.4)	0.005
Urine protein, *n* (%)	21 (10.5)	12 (14.0)	0.524
Urine leukocytes, *n* (%)	70 (35.0)	27 (31.4)	0.650
Urine specific gravity, mean (SD)	1.0 (0.0)	1.0 (0.0)	0.463
GOT, U/L, mean (SD)	29.4 (57.5)	25.2 (18.8)	0.356
Glutamic pyruvic transaminase, U/L, mean (SD)	25.7 (42.7)	24.2 (24.4)	0.708
Gamma-glutamyl transferase, U/L, mean (SD)	47.2 (66.6)	49.3 (87.8)	0.840
Alkaline phosphatase, U/L, mean (SD)	93.7 (69.4)	92.1 (79.2)	0.870
Total bilirubin, μmol/L, mean (SD)	10.8 (8.0)	11.7 (9.0)	0.396
Total protein, g/L, mean (SD)	69.9 (7.3)	67.8 (7.5)	0.037
Albumin, g/L, mean (SD)	39.2 (5.2)	37.7 (4.9)	0.020
Serum creatinine, μmol/L, mean (SD)	83.0 (64.5)	75.6 (22.9)	0.156
Blood urea nitrogen, mmol/L, mean (SD)	6.6 (5.0)	6.2 (3.0)	0.413
Serum uric acid, μmol/L, mean (SD)	324.0 (129.0)	343.5 (112.0)	0.200
Globulin, g/L, mean (SD)	30.7 (6.1)	30.1 (5.4)	0.481
Blood glucose, mmol/L, mean (SD)	6.0 (2.2)	6.0 (2.1)	0.931
APTT, s, mean (SD)	28.6 (12.7)	26.7 (6.2)	0.095
D-dimer, μg/L, mean (SD)	4218.1 (13590.5)	9973.1 (17662.0)	0.008
Fibrinogen, g/L, mean (SD)	3.6 (1.5)	3.6 (1.3)	0.700
Thrombin time, s, mean (SD)	12.6 (7.8)	12.1 (1.5)	0.348
Prothrombin activity, %, mean (SD)	12.6 (7.8)	12.6 (5.4)	0.982
Prothrombin time, s, mean (SD)	12.6 (7.8)	12.1 (1.6)	0.382
International normalized ratio, mean (SD)	1.1 (0.7)	1.0 (0.1)	0.348
Erythrocyte sedimentation rate, mm/h, mean (SD)	26.7 (28.6)	20.3 (18.9)	0.025
Surgery, n (%)	144 (72.0)	67 (77.9)	0.371
Bed rest duration, day, mean (SD)	12.0 (9.1)	15.0 (21.2)	0.210

VTE, venous thromboembolism; BMI, body mass index; GOT, glutamic-oxaloacetic transaminase; APTT, activated partial thromboplastin time.

*Polygenic risk criteria: Low risk: both loci are wild-type (MTHFR C/C and PAI-1 5G/5G); Intermediate risk: one locus is heterozygous mutant (C/T or 4G/5G), and the other remains wild-type; High risk: both loci are heterozygous mutant (MTHFR C/T and PAI-1 4G/5G), or at least one locus is homozygous mutant (MTHFR T/T or PAI-1 4G/4G), regardless of the status of the other locus.

### Feature selection

After removing features with more than 40% missing values, 34 features remained, including: polygenic risk, gender, race, age, BMI, smoking history, alcohol consumption history, VTE history, comorbidities, urine protein, urine leukocytes, urine specific gravity, glutamic-oxaloacetic transaminase (GOT), glutamic pyruvic transaminase, gamma-glutamyl transferase, alkaline phosphatase, total bilirubin, total protein, albumin, serum creatinine, blood urea nitrogen, serum uric acid, globulin, blood glucose, activated partial thromboplastin time (APTT), D-dimer, fibrinogen, thrombin time, prothrombin activity, prothrombin time, international normalized ratio, erythrocyte sedimentation rate, surgery, bed rest duration.

**Figure 3:**
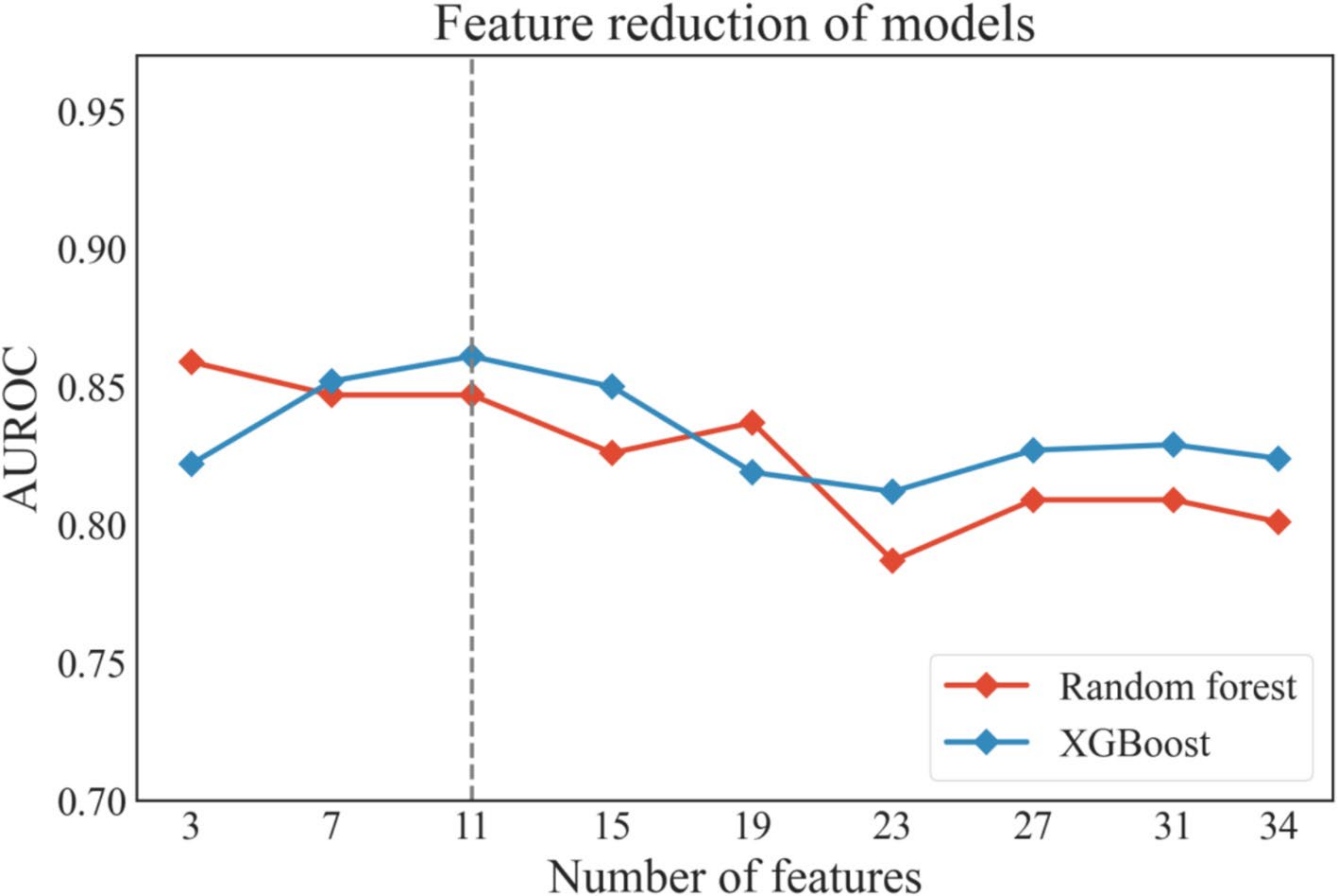
The performance of the models during recursive feature elimination. XGboost, eXtreme gradient boosting.

Based on Random Forest model and XGBoost model, Recursive Feature Elimination (RFE) was applied to select the optimal feature subset, as shown in Figure 3. Among the two models, a feature subset with 11 features achieved the optimal performance on the XGBoost model. The 11-feature subset consisted of D-dimer, erythrocyte sedimentation rate, blood glucose, urine specific gravity, serum creatinine, urine leukocytes, VTE history, activated partial thromboplastin time (APTT), bed rest duration, gender and age. Due to without differences (*p* < 0.1) between VTE and non-VTE patients, five features, including bed rest duration, blood glucose, urine specific gravity, serum creatinine and urine leukocytes, were eliminated, as shown in Table 1. We also substituted gender with comorbidities that showed stronger association with the outcome, as shown in Figure 1. Given that previous literature has indicated that the genetic polymorphisms MTHFR (C677T) and PAI-1(4G/5G) are associated with an increased risk of VTE (21–23), the polygenic risk based on these two polymorphic sites were included as features in the model construction. Although statistical analysis did not show a significant difference between the VTE and non-VTE groups, these genetic characteristics still hold important value in VTE risk assessment. Additionally, according to the literature, bed rest duration is also a critical factor influencing the occurrence of VTE.

Finally, 8 features were selected to construct the model: age, VTE history, comorbidities, APTT, D-dimer, erythrocyte sedimentation rate, bed rest duration, and polygenic risk.

### Model construction and performance comparison

**Figure 4:**
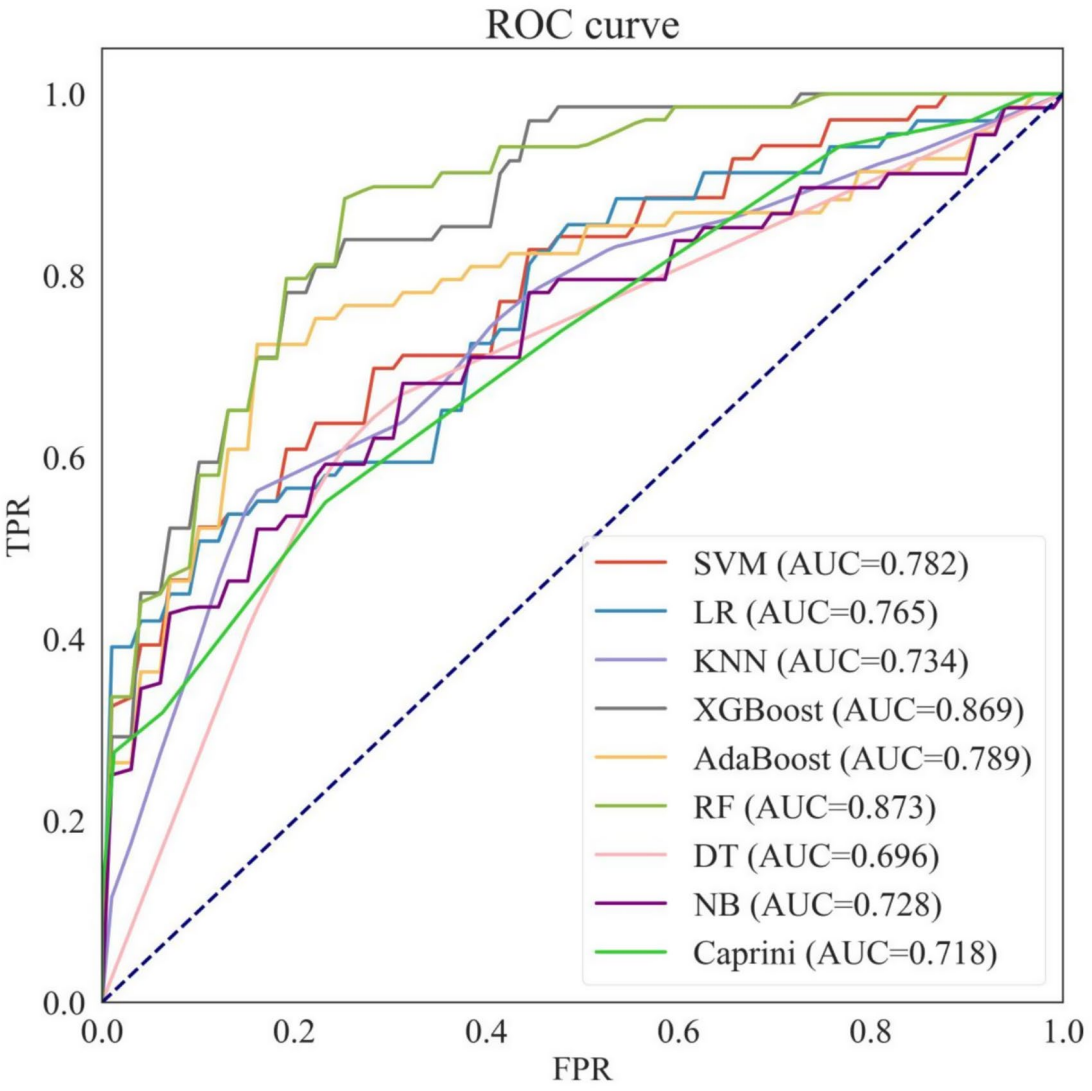
Receiver operating characteristic curves of eight machine learning models and caprini score on the training set. NB, naive bayes; KNN, k-nearest neighbors; SVM, support vector machines; LR, logistic regression; DT, decision tree; AdaBoost, adaptive boosting; XGboost, eXtreme gradient boosting; RF, Random Forest; AUC, area under the curve.

Based on the training dataset, eight machine learning prediction models were constructed, including SVM, LR, KNN, XGBoost, AdaBoost, RF, DT, and NB. Table 2 shows the average performance of these models under five-fold cross-validation, and the ROC curves are presented in Figure 4.

**Table 2:** Performance of eight machine learning models and caprini score on the training set.

Model	Accuracy	Sensitivity	Specificity	Precision	F1 Score	AUROC
NB	0.719	0.464	0.83	0.53	0.484	0.728
KNN	0.675	0.623	0.698	0.472	0.535	0.734
SVM	0.763	0.448	0.898	0.696	0.517	0.782
LR	0.794	0.434	0.95	0.785	0.552	0.765
DT	0.719	0.636	0.755	0.533	0.576	0.696
AdaBoost	0.776	0.693	0.811	0.619	0.65	0.789
XGBoost	0.785	0.724	0.811	0.621	0.668	0.869
RF	0.777	0.697	0.812	0.607	0.643	0.873
Caprini	0.447	0.942	0.233	0.348	0.508	0.718

NB, naive bayes; KNN, k-nearest neighbors; SVM, support vector machines; LR, logistic regression; DT, decision tree; AdaBoost, adaptive boosting; XGboost, eXtreme gradient boosting; RF, Random Forest.

Among the eight models, Random Forest (RF) and XGBoost outperformed the others, achieving the best predictive performance with AUROCs of 0.873 and 0.869, respectively, making them the two best-performing predictive models selected for further hyperparameter tuning.

Additionally, when comparing the eight machine learning models with the traditional Caprini score, seven of the models (except Decision Tree) achieved higher AUROCs than the Caprini score. Moreover, the accuracy, specificity, and precision of all eight machine learning models exceeded those of the Caprini score.

### Hyperparameter tuning and validation of the models

**Figure 5:**
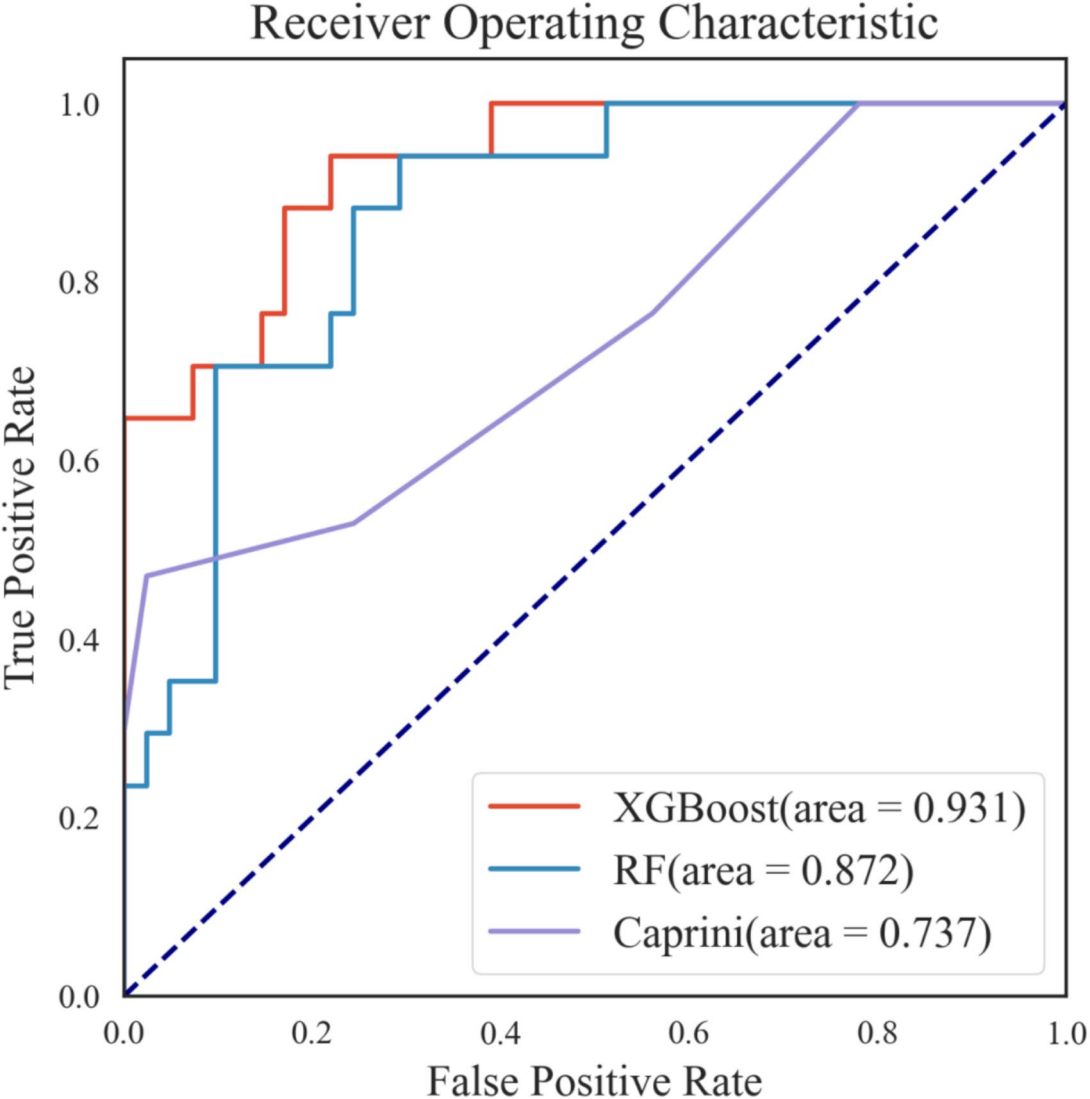
Receiver operating characteristic curves of the two best-performing machine learning models and caprini score on internal validation set.

The combination of different parameters can directly impact the predictive ability, generalization performance, and practical applicability of a model. In this study, the optimal hyperparameter combination was obtained through random search and manual fine-tuning, resulting in the following settings of the XGBoost model: subsample of 0.72, n_estimators of 50, min_child_weight of 1, max_depth of 6, learning_rate of 0.074, gamma of 0.25, and colsample_bytree of 0.5, and the following settings of the Random Forest model: n_estimators of 300, min_samples_split of 2, max_features of 3, max_depth of 5, and bootstrap of True. The discriminative ability of the two model on the test set was shown in Figure 5, revealing that the XGBoost model had the best predictive performance. So, the XGBoost model was selectd as the final model.

**Figure 6:**
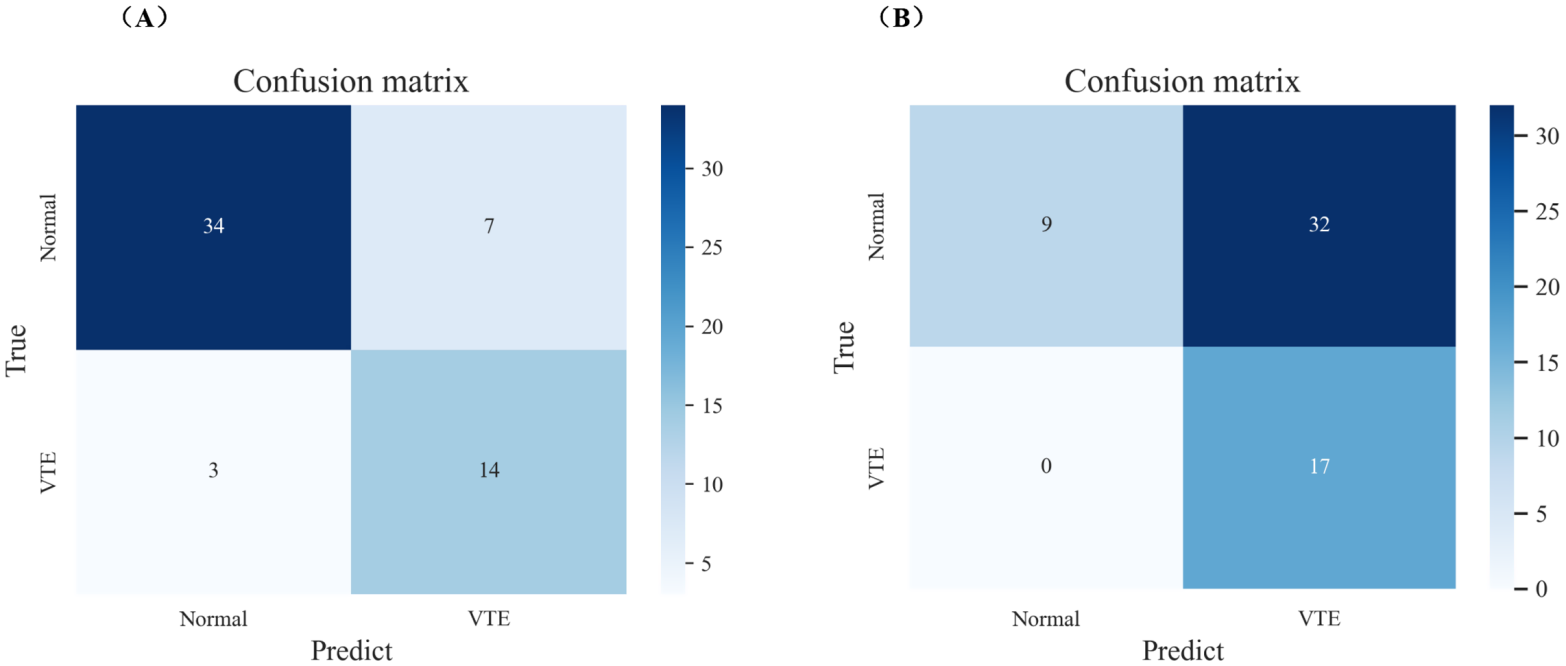
The performance of the final model and caprini score on internal validation set. **(A)** Confusion matrix of the final model on internal validation set. **(B)** Confusion matrix of caprini score on internal validation set.

The test set consisted of 58 samples, including 41 negative cases and 17 positive cases. The final model performed well on the test set, as shown in Figures 5, 6A. The final model achieved an accuracy of 0.828, sensitivity of 0.824, specificity of 0.829, precision of 0.667, F1 score of 0.737, and AUROC of 0.931. Among the 41 negative samples, 34 were correctly predicted, while 14 of the 17 positive samples were correctly predicted.

Additionally, the final model outperformed the Caprini score on the test set, as detailed in Figures 5, 6. The Caprini score achieved an AUROC of 0.737, correctly predicting 9 out of 41 negative samples and 17 out of 17 positive samples. The Caprini score tended to overestimate low-risk cases as high risk for VTE.

### Model explanation

**Figure 7:**
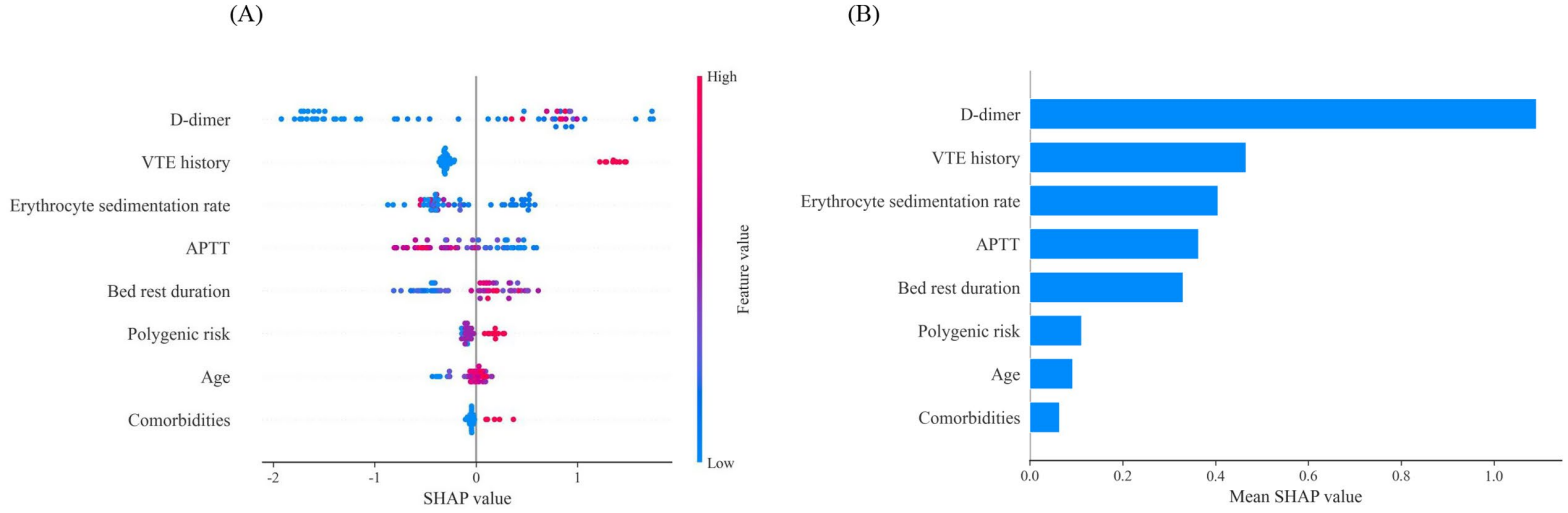
Global interpretability for the final model prediction by SHAP method. **(A)** SHAP summary plot of 8 features included in the final prediction model for VTE. The horizontal axis represents the SHAP values, and the vertical axis represents the features. Each point corresponds to a sample, with the color of the points indicating the magnitude of the feature values: red represents higher feature values, while blue represents lower feature values. A positive SHAP value indicates an increased risk of VTE. Additionally, a positive correlation is shown when higher feature values result in larger SHAP values. **(B)** SHAP summary plot of 8 features ranked by the mean absolute SHAP values across all samples, representing the average impact of each feature on the prediction of VTE. VTE, venous thromboembolism; APTT, activated partial thromboplastin time.

Since clinicians often find it difficult to accept predictive models that are not directly interpretable or understandable, the SHAP method was employed to explain the output of the final model by quantifying each feature’s contribution to the prediction. The main advantage of SHAP lies in its ability to provide both global and local interpretability. Global interpretation highlights the most influential features in the model’s decision-making process. Figures 7A,B present SHAP summary plots, where the SHAP mean value represents each feature’s contribution to the model predictions, ranked in descending order of importance. The order of importance is as follows: D-dimer, VTE history, erythrocyte sedimentation rate, APTT, bed rest duration, polygenic risk, age, and comorbidities.

**Figure 8:**
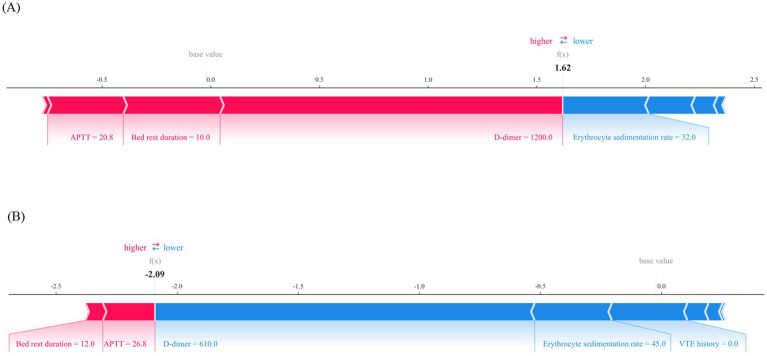
Local interpretability for the final prediction model by SHAP method. **(A)** Force plot of a patient with VTE, APTT, bed rest duration and D-dimer are major features contributing to a higher predicted risk of VTE. **(B)** Force plot of a patient without VTE, D-dimer, erythrocyte sedimentation rate and VTE history are major features contributing to a lower predicted risk of VTE. APTT, activated partial thromboplastin time; VTE, venous thromboembolis.

The model predicts outcomes for specific individuals by assigning a SHAP value to each feature. As shown in Figures 8A,B, predictions for negative and positive individuals are visualized. The length of the bars represents the magnitude of the feature’s impact on the final prediction, with red bars indicating a positive contribution and blue bars indicating a negative contribution. For positive individuals, APTT, bed rest duration, and D-dimer drive the model to predict VTE; for negative individuals, erythrocyte sedimentation rate, VTE history, and D-dimer drive the model to predict non-VTE.

## Discussion

In this study, we extracted data from the electronic medical record system of orthopedic inpatients and applied eight machine learning algorithms to build predictive models for the risk of VTE. The goal was to identify the optimal model for predicting the occurrence of VTE. Additionally, to validate the performance advantages of machine learning methods, we also used the traditional Caprini score to predict VTE risk in the same group of patients. A comparative analysis of the results revealed that the performance of the Caprini score model was significantly lower than the optimal XGBoost model. Specifically, the Caprini score model has high sensitivity but very low specificity, meaning a significant number of patients are misclassified as having a VTE risk, resulting in a high false positive rate. Even though the final AUROC is 0.737, the Caprini score performs poorly in predicting negative results. In contrast, the evaluation metrics of the XGBoost model were as follows: sensitivity of 0.824, specificity of 0.829, and an AUROC of 0.931, indicating that the XGBoost model demonstrated superior performance in identifying VTE risk, providing more accurate and effective support for clinical decision-making.

In addition, the innovation of this study lies in incorporating genetic factors related to VTE in the Chinese population into the predictive model, combined with clinical phenotype data to construct a comprehensive risk prediction model. Previous studies have shown that Factor V Leiden (rs6025) and prothrombin G20210A mutations are typical genetic risk factors for VTE (24, 25); however, these mutations are relatively rare in the Chinese population (26). Therefore, this study selected the MTHFR (C677T) and PAI-1(4G/5G) variants, which are more common in the Chinese population, for investigation (21–23). Mutations in the MTHFR gene can lead to hyperhomocysteinemia, which in turn causes endothelial injury, increasing the risk of VTE (27, 28). Variations in the PAI-1 gene inhibit the fibrinolytic system, resulting in fibrinolysis dysfunction, which becomes an important trigger for thrombosis (29, 30). By incorporating the genetic variations of MTHFR and PAI-1 into the model, this study aims to further improve the accuracy and clinical applicability of the VTE risk prediction model.

However, this study has several limitations. First, the relatively small sample size may not fully capture the diverse risk characteristics of all orthopedic inpatients, which limits the broader applicability of the findings. Second, the study was conducted at a single center and lacks multi-center validation, which could affect the model’s generalizability across different clinical settings. Third, due to substantial missing data for some variables, these features could not be included in the analysis, which may restrict the model’s ability to fully elucidate the complex biological mechanisms underlying venous thromboembolism (VTE). As such, while the findings of this study show promising clinical potential, caution is necessary when interpreting the results. Future research should focus on larger, multi-center cohort studies and integrate additional biomarkers to address these limitations and enhance the robustness of the model. The ultimate goal is to integrate the model into clinical decision support systems, enabling real-time prediction and intelligent warning of VTE risk based on dynamic clinical data.

In conclusion, this study successfully developed a machine learning-based VTE risk prediction model for orthopedic inpatients. The model demonstrated strong predictive performance on both the training and testing datasets, highlighting its potential for early identification of high-risk patients in clinical practice. These findings underscore the importance of integrating advanced analytical methods into clinical risk assessment, laying the foundation for personalized preventive strategies in VTE management. Future research should focus on validating the model in larger, more heterogeneous populations and exploring the integration of additional clinical and molecular data to further enhance prediction accuracy and utility, improve model generalizability, and ultimately benefit patients, advancing the development of personalized medicine.

## Data Availability

The original contributions presented in the study are included in the article/supplementary material, further inquiries can be directed to the corresponding author.
